# Economic Aspects of a Concrete Floating Offshore Wind Platform in the Atlantic Arc of Europe

**DOI:** 10.3390/ijerph16214122

**Published:** 2019-10-25

**Authors:** Eugenio Baita-Saavedra, David Cordal-Iglesias, Almudena Filgueira-Vizoso, Laura Castro-Santos

**Affiliations:** 1 Escola Politécnica Superior, Universidade da Coruña, Esteiro, 15471 Ferrol, Spain; eugenio.baita@udc.es (E.B.-S.); david.cordal@udc.es (D.C.-I.); 2 Departamento de Química, Escola Politécnica Superior, Universidade da Coruña, Esteiro, 15471 Ferrol, Spain; almudena.filgueira.vizoso@udc.es; 3 Departamento de Enxeñaría Naval e Industrial, Escola Politécnica Superior, Universidade da Coruña, Esteiro, 15471 Ferrol, Spain

**Keywords:** floating offshore wind, concrete wind platform, economic feasibility, IRR, NPV, LCOE

## Abstract

The objective of this paper is to examine the economic aspects of a concrete offshore wind floating platform in the Atlantic Arc of Europe (Portugal and Spain). The life-cycle cost of a concrete floating offshore wind platform is considered to calculate the main economic parameters that will define the economic feasibility of the offshore wind farm. The case of study is the concrete floating offshore wind platform Telwind^®^, a spar platform with a revolutionary way of installing using a self-erecting telescopic tower of the wind turbine. In addition, the study analyses thirteen locations in Spain and twenty in Portugal, including the Atlantic islands of both countries. Results indicate that the economically feasible location to install a concrete offshore wind farm composed of concrete platforms is the Canary Islands (Spain) and Flores (Portugal).

## 1. Introduction

The global economy is committed to reducing greenhouse gas emissions [1]. To this end, policies to reduce them are being promoted. The 2015 Paris agreement [2] highlights the importance of cross-border cooperation in environmental matters between member states, including Spain and Portugal. The priority objective is to reduce greenhouse gases by 20% compared to 1990 and reach 80% by 2050 [3]. In recent years, the European Union has made considerable efforts to increase electricity generation through renewable sources. The percentage of renewable energies in the final electrical consumption has doubled from 8.5% in 2004 to 17% in 2016 [4]. Although the EU, as a whole, is on its way to achieve the objectives of H2020, some member states, including Spain and Portugal, will have to make additional efforts to meet their obligations.

In 2008, the first generation of commercial marine energy systems was introduced, with the first platforms located in the United Kingdom and Portugal (Seagen and Pelamis, respectively) [5]. With this, there are currently three main sorts of energy devices from which energy can be produced commercially (wind, waves, and tides) in marine regions. Each of these technologies can be presented individually or as a combination of several, depending on the characteristics of each area, and they are clean energies both in the sense that they do not produce greenhouse gases [6], but also in the visual aspect, which is minimal compared to other energy sources [7].

Within these oceanic energy sources, offshore wind energy can become one of the main sources of the future European energy system. It is one of the basic energies to achieve the objectives of climate change policies [8]. Wind Europe expects 150 GW of wind capacity to be achieved in 2030 that would supply 14% of Europe’s electricity demand. Analyzing wind power, it can be seen that offshore power has augmented by 19.7% in the last 10 years [9], being therefore, one of the renewable energies with the greatest potential [10]. In addition to this, as stated by Noori et al., offshore wind turbines produce 48% less greenhouse gas emissions per kWh of produced electricity than onshore wind turbines.

It was also found that the higher the capacity of the wind turbine, the lower the environmental impact.

According to Chipindula et al. and Shifeng Wang, Wang et al., the life-cycle GHG (Greenhouse Gas) emission intensity is 0.082 kg CO_2_-equivalent (eq)/Mega-joule (MJ) and 0.130 kg CO_2_-eq/MJ for an onshore and offshore wind turbine, respectively. Both onshore and offshore energy have lower GHG emissions than coal plants (Lei Xu et al. 2018).

Within the marine wind energy, there are two types: Fixed structures (up to 50 m of depth) and floating platforms (over 50 m of depth). Spain and Portugal have very deep continental platforms in areas very close to the coast, which makes it necessary to install floating platforms. Within these, the only commercial ones (Windfloat of Principle Power [11] and Hywind of Statoil [12,13]) are made entirely of steel: It is a very common material in maritime civil engineering, which is resistant to corrosion, does not have high maintenance costs, and is easy to obtain. However, materials such as concrete have a number of characteristics that make it interesting to analyze platforms built with this material. Therefore, this article is focused on the economic analysis of an offshore concrete floating wind platform, a 10 MW Telwind^®^ [14] (mast platform with a revolutionary form of installation), in the Atlantic Arc of Europe. The cost of the life cycle [15,16,17] will be considered to determine the main economic parameters that will determine the economic viability of the park: Cost of the life cycle, levelized cost of energy (LCOE), net present value (NPV), and internal rate of return (IRR). The study analyzed thirteen locations in Spain [17] (the Iberian Peninsula and the Canary Islands) and twenty in Portugal [18] (the Iberian Peninsula, the Azores Islands, and the Madeira Islands) [19]. The results show which are the most suitable areas, in economic terms, to install a marine wind farm of concrete.

## 2. Materials and Methods

The work methodology of this study is founded on life-cycle cost methodology for floating offshore wind steel platforms [15], but applying the procedure to concrete structures, as defined in the case study.

### 2.1. Determination of Wind Production

**Figure 1:**
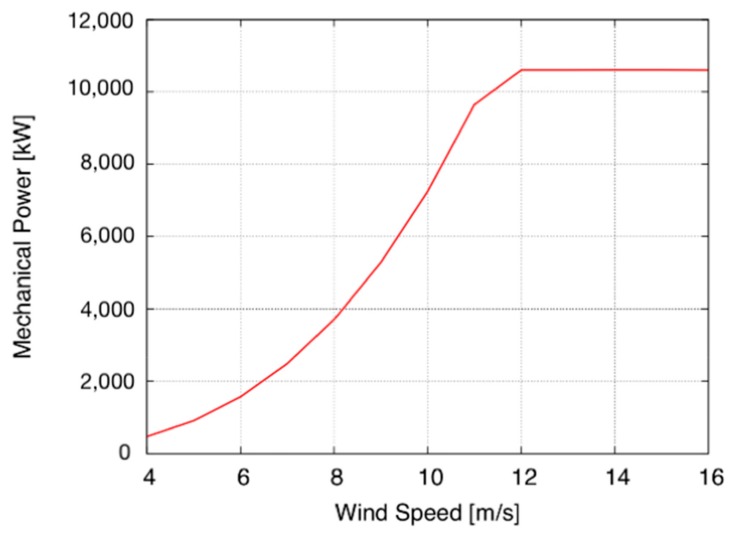
Power curve of the DTU 10MW wind turbine.

The annual produced energy depends on the characteristic power curve of the turbine and the wind speed distribution function of each location. The DTU 10 MW wind turbine with a constant capacity of 10 MW has been selected for the analysis [20]. Figure 1 shows the power curve of this turbine.

In this study, the Weibull distribution is used to characterize wind behavior. The Weibull distribution is a continuous probability distribution that is usually used to define the variation of wind speed at a given location, and thus, describe the behavior of the wind through parameters that define its probability function [21,22].

(1) $$f\left(v\right)=\left(\frac{k}{c}\right)\cdot {\left(\frac{v}{c}\right)}^{k-1}\cdot e^{{\left[-\left(\frac{v}{c}\right)\right]}^{k}}$$

where *k*: shape factor Weibull distribution, *c*: scale factor Weibull distribution, *v*: wind speed.

Data of the parameters of each location are necessary. The energy produced is calculated considering Equation (2), where $P_{PC}\left(v\right)$ is the power curve of the offshore wind turbine and $p_{Weibull}\left(v,k,c\right)$ is the probability function.

(2) $$E=\int_{0}^{v_{out}}P_{PC}\left(v\right)\cdot f\left(v\right)\;$$

### 2.2. Life Cycle Cost Assessment

**Figure 2:**
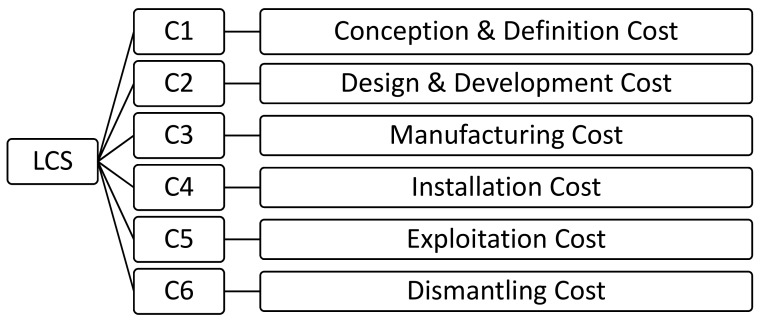
Life cycle system (LCS) phases. Source: [15].

Figure 2 shows the phases of the LCS (life cycle system) of a floating offshore wind farm.

In this way, the total cost system of the life cycle of the farm (LCS) is obtained from (3) [15]

(3) $$LCS=C_{1}+C_{2}+C_{3}+C_{4}+C_{5}+C_{6}$$

The manufacturing costs and the installation costs are some the most relevant costs in terms of the differences between the traditional steel floating offshore wind platforms and the concrete one considered in this study.

To analyze manufacturing and installation costs, each of the components that constitute a floating offshore wind farm has been considered and studied separately as a subphase of costs.

The subphases of costs considered for *C*_3_ and *C*_4_ have been the generator, the floating platform, the mooring, the anchoring, and the electric system. In this way, total manufacturing and installation costs have been obtained as the sum of all subphases.

Equation (4) allows to obtain the transport costs of an offshore wind turbine (*C*_41_).

(4) $$C_{41}=C_{411}+C_{412}$$

where:

- *C*_411_: Onshore installation and precommissioning cost (€).
- *C*_412_: Offshore commissioning cost (€).

The main costs that may affect each operation have been considered. For onshore installation and precommissioning, a self-propelled modular transporter (SPMT) is used to carry out the transport operations of the turbine components.

(5) $$C_{411}=N_{turbine}\cdot \left(C_{nacelle}+SPMT_{blades}+SPMT_{nacelle}+C_{other}\right)$$

where:

- *N_turbine_*: Number of turbines.
- *C_nacelle_*: Nacelle assembly cost of a 10 MW turbine (€).
- $SPMT_{bladess}$: *SPMT* for concrete blades cost of a 10 MW turbine (€).
- $SPMT_{nacelle}$: *SPMT* for nacelle cost of a 10 MW turbine (€).
- *C_other_*: Other costs of a 10 MW turbine (€).

For offshore commissioning, the costs of technicians and specialized personnel have been considered.

(6) $$C_{412}=N_{turbine}\cdot \left(N_{tech}\cdot r_{daily}\cdot n_{days}\right)$$

where:

- *N_tech_*: Number of offshore commissioning technicians.
- *r_daily_*: Offshore commissioning technician daily rate (€/days).
- *n_days_*: Time required for offshore commissioning (days).

As in the equations shown, many other input data are necessary to complete the entire cost assessment.

Exploitation costs (*C*_5_) consist of insurance (obtained from 1% of *C*_1_ + *C*_2_ + *C*_3_ + *C*_4_), administration and operations costs (data acquisition cost, SAP (systems, applications, products, and data processing) and maritime coordination cost, meteorological prediction cost), maintenance costs (turbine, export cable, grid connection, and substructure maintenance, inter-array cable survey) and logistics both onshore and offshore.

In order to calculate decommissioning costs (*C*_6_), percentages have been used according to the costs of the dismantled material, as shown in Table 1.

### 2.3. Determination of Economic Parameters

The main economic indicators used in this study that will determine the economic feasibility of the farm are: The internal rate of return (IRR), the net present value (NPV), and the levelized cost of energy (LCOE).

The NPV represents the present value of future cash flows that will be created or are the result of a specific investment.

This is a dynamic criterion since it takes into account the updating of cash flows, both their amount and the time when they are obtained, in order to homogenize them over time. The result is obtained in absolute terms of the monetary units.

The NPV was calculated as follows:

(7) $$NPV=-CF_{0}+\frac{CF_{1}}{1+k}+\frac{CF_{2}}{{\left(1+k\right)}^{2}}+\ldots +\frac{CF_{n-1}}{{\left(1+k\right)}^{n-1}}+\frac{CF_{n}}{{\left(1+k\right)}^{n}}$$

where:

- *CF*_0_: Initial investment (€).
- *CF_n_*: Cash flow in time n (€).
- *n*: Project lifetime (year).
- *k*: Discount rate.

The criterion of acceptance around the NPV is that it is positive, and, among several projects, it will be more convenient than that with a higher NPV.

The internal rate of return (IRR) measures the expected future returns for a given investment, and implies the supposed case of an opportunity to invest. It is used, together with the NPV, as a criterion for deciding between the acceptance and rejection of an investment project. The higher the IRR, the greater the profitability.

The IRR corresponds to the discount rate (*k*) that the NPV makes zero. Therefore, it is defined with the following expression:

(8) $$0=-CF_{0}+\frac{CF_{1}}{1+k}+\frac{CF_{2}}{{\left(1+k\right)}^{2}}+\ldots +\frac{CF_{n-1}}{{\left(1+k\right)}^{n-1}}+\frac{CF_{n}}{{\left(1+k\right)}^{n}}$$

The levelized cost of energy (LCOE) is the most representative indicator to calculate the cost of wind energy production. The cost components that has been estimated to calculate this indicator are the initial investment and the operation and maintenance costs. The annual value of the LCOE is estimated considering (9):

(9) $$LCOE=\frac{\sum_{t=0}^{N_{farm}}\frac{LCS_{t}}{{\left(1+r\right)}^{t}}}{\sum_{t=0}^{N_{farm}}\frac{E_{t}}{{\left(1+r\right)}^{t}}}$$

where:

- *LCOE*: *LCOE* of wind energy (€/MWh).
- $E_{t}$: Energy produced by the farm (MWh/year).
- $N_{farm}$: Life-cycle of the farm (years).

## 3. Configuration of the Offshore wind Farm Platform, TELWIND

**Figure 3:**
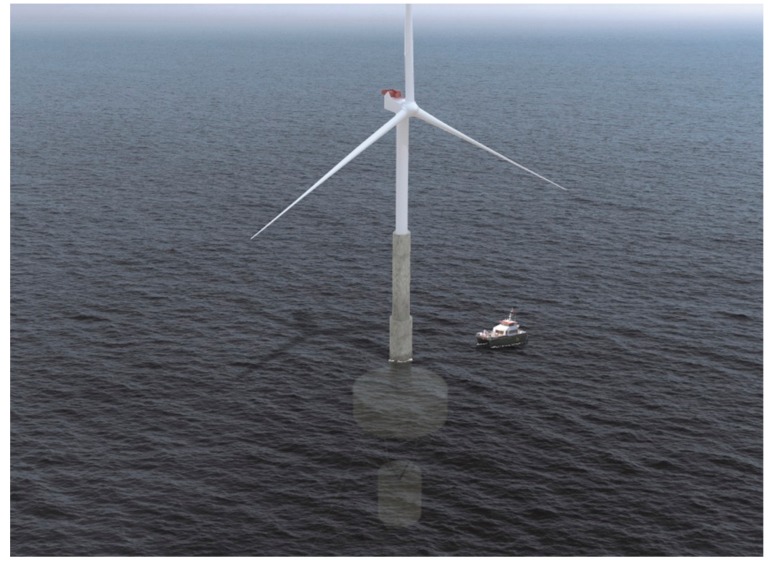
Telewind^®^ platform. Source: Figure courtesy of Esteyco [15].

The floating offshore wind platform studied in this work is the Telewind^®^, an evolved concrete spar floating offshore wind platform designed by the Spanish enterprise Esteyco (see Figure 3). The offshore wind farm studied has a capacity of 500 MW.

**Figure 4:**
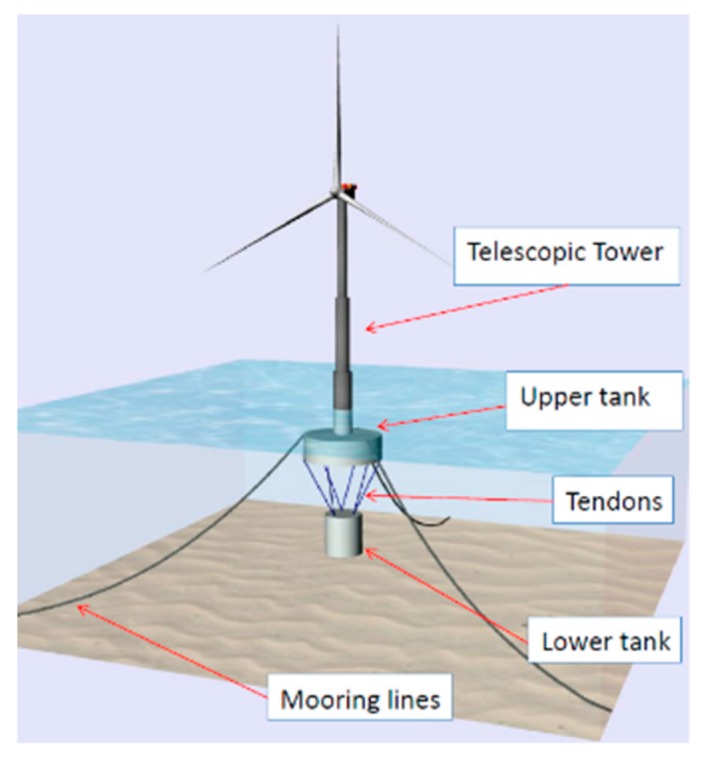
Main parts of the Telewind^®^ structure. Source: Figure courtesy of Esteyco [23,24,25].

The main components of the Telewind^®^ platform are (see Figure 4): Telescopic tower, upper tank, tendons, lower tank, and mooring lines.

**Figure 5:**
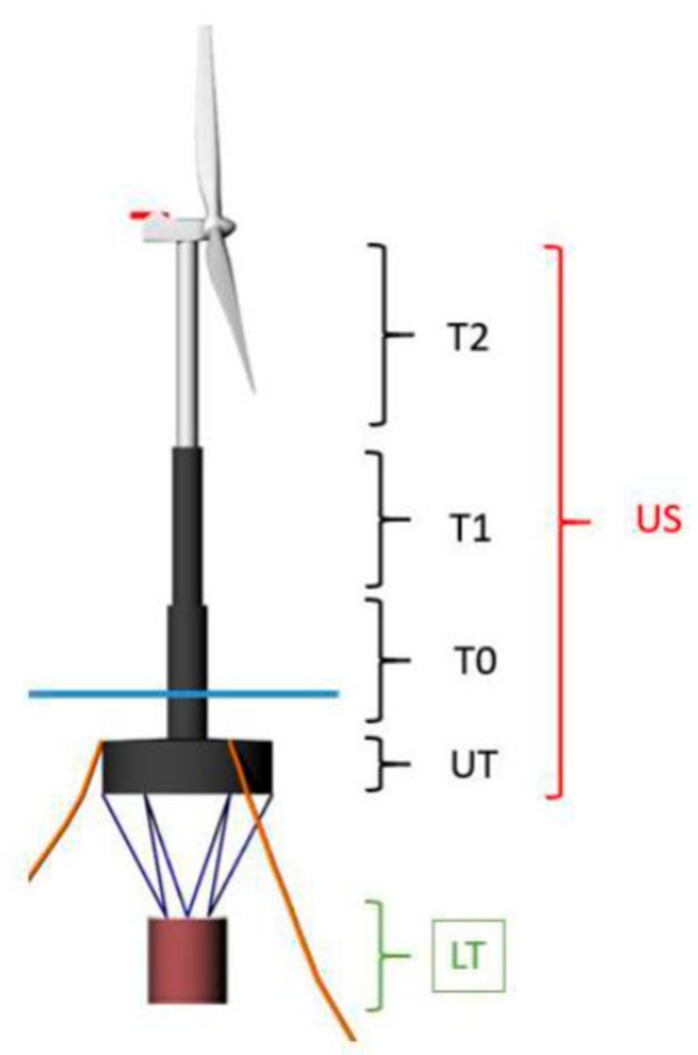
Parts of the Telewind^®^ platform. Source: Figure courtesy of Esteyco.

The Telewind^®^ platform is composed of two parts (see Figure 5): The upper structure (US) and the lower tank (LT). Moreover, the upper structure is composed of the upper tank (UT), tower 0 (T0), tower 1 (T2), and tower 2 (T2). T1, T0, UT, and LT are built in concrete and T2 is built in steel for the Arcwind project. The description of the main parts of the Telewind^®^ platform is as follows (from courtesy of Esteyco):

- Wind turbine generator (WTG): It is worth to note that only the nacelle, hub, and blades are part of WTG.
- Tower (TW): Telescopic concrete tower divided into several sections with variable thickness. The telescopic tower is divided into three sections (T0, T1, T2). It should be noted that the upper section (T2) of the tower is completely made of steel, while T0 and T1 are made of concrete. The T0 is partially submerged and it is part of the “platform hull”, it means it is designed according to accepted offshore industry design criteria taking into account the hydrostatic pressures, waves, and currents plus the contribution due to wind forces.
- Upper tank (UT): Wet part of the platform which comprises the cylindrical base. It provides buoyancy in excess to guarantee the stability of the system. Although it is not really relevant at this stage, the base is internally compartmented to minimize free surface effects, sloshing and to improve the structural strength. Note that the submerged part of the T0 is part of the tower (TW).
- Upper structure (US): In order to simplify the nomenclature, US comprises the WTG + TW + UT.
- Lower tank (LT): Suspended body filled with a combination of solid and water ballast when it is in place. For installation purposes, the lower tank is water ballasted until it gets the final location.
- Tendon suspension system: Cable connections between the UT and the LT. Tendons may be made of steel or synthetic fibers. The final configuration of lines and its connection with UT and LT will be defined in a later stage of the project.
- Mooring system: The mooring system is comprised by anchors, mooring lines, connectors, and links. It is composed of several catenary mooring lines (chain, fibers or mixed systems). The final arrangement will be defined later in detail phases.

The main characteristics of the Telewind^®^ platform are shown in Table 2.

## 4. Study Locations

**Figure 6:**
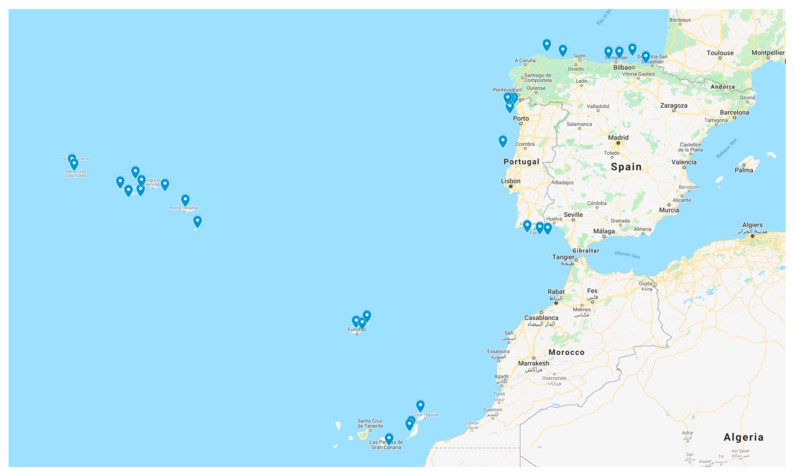
Locations of the study. Source: Own elaboration.

The case of the study is focused on two countries of the Atlantic Arc of Europe (see Figure 6): Spain and Portugal. The offshore wind sector will have great importance in these countries, which have been pioneers in two different aspects of the offshore wind: Floating offshore wind building using shipyards and floating offshore installation, respectively. Portugal installed the second floating platform in the world, the WindFloat semisubmersible platform, years ago. Nowadays the WindFloat Atlantic project (Windplus) is being carried out as the first floating offshore wind farm in the Iberian Peninsula. Additionally, Navantia Fene (in A Coruña province, Galicia region of NW of Spain) built five Hywind spar platforms for the Hywind Scotland farm, the first commercial floating offshore wind farm in the world, and it is building five platforms for the Kinkardine farm, which will be installed in the UK [26,27,28].

In this context, thirteen locations have been considered in Spain: Nine in the Iberian Peninsula (A Guarda-Baiona 1, A Guarda-Baiona 2, Ribadeo, Navia, San Vicente de la Barquera, Santander, Bilbao, Mutriku, Huelva) and four in the Canary Islands (Lanzarote, Fuerteventura 1, Fuerteventura 2, Gran Canaria). In addition, twenty locations have been considered in Portugal: Seven in the Iberian Peninsula (Vianao do Castelo 1, Viana do Castelo 2, Póvoa do Varzim, Porto, Figueira da Foz, Algarve-Albufeira, Algarve-Faro), ten in the Azores Islands (Flores 1, Flores 2, Faial, Pico 1, Pico 2, Sao Jorge, Graciosa, Terceira, Sao Miguel, Santa María) and three in the Madeira Islands (Sao Vicente-Santana, Porto da Cruz-Caniçal, Porto Santo). Their coordinates are in Table 3.

**Figure 7:**
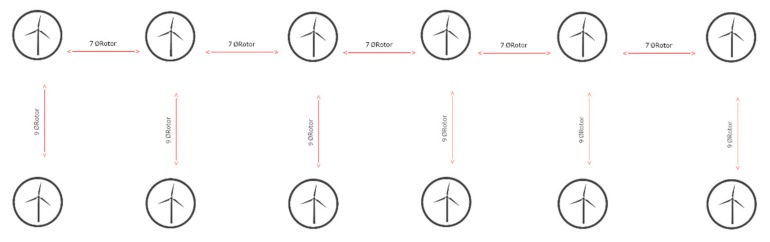
Structure of a wind farm. Source: own elaboration.

On the other hand, it is important to consider the distance farm–shore (m), the distance farm–onshore facilities (m) and the bathymetry (m) of the location, which are shown in Table 4, and the separation between wind turbines (Figure 7).

The distance between wind turbines is seven times the diameter of the rotor of the wind turbine and the distance between lines of wind turbines is nine times the diameter of the rotor of the wind turbine, as shown in Figure 7.

On the other hand, three alternatives have been considered regarding the electric tariff considered (50, 100, and 150 €/MWh) (see Table 5). It is important to notice that the electric tariff changes depending on the country selected. Nowadays, there is no specific electric tariff regulation for floating offshore wind in Spain. While the electricity tariffs in Spain and Portugal are different, the range of tariffs in each country should be still stated. This is the reason why these alternatives have been taken into account.

## 5. Results

Once the case study and the different alternatives have been defined, the calculation methodology explained above has been applied to obtain the life cycle cost and the economic parameters. The economic parameters LCOE, NPV, and IRR have been obtained in order to compare the locations proposed.

**Figure 8:**
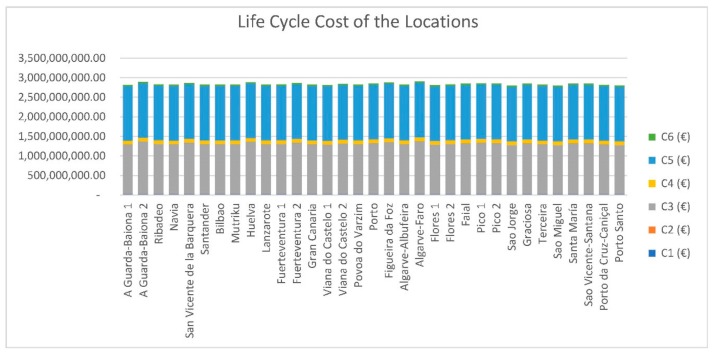
Life-cycle cost of the locations.

Figure 8 shows the life cycle costs of each proposed alternative. The most important phases of the life cycle of a floating offshore wind farm are the manufacturing cost and the operation and maintenance costs. The alternative with a lower life cycle cost is Sao Jorge, which is due to the strategic conditions of the location (1 km distance from shore).

The manufacturing costs considered are almost the same, since the size of the farm and the number of turbines is constant. The maintenance costs may vary depending on the distance to the coast, but the differences between the locations studied are not very large, ranging between 8 and 35 km.

**Figure 9:**
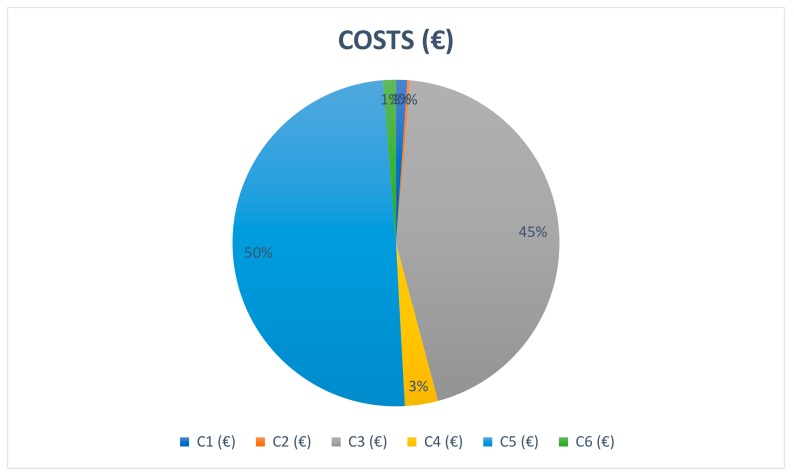
Life-cycle cost of São Jorge.

The relevance of each life cycle phase is shown in Figure 9 for the São Jorge location. The most important costs of a floating offshore windfarm are exploitation cost and manufacturing cost, reaching 50% and 45%, respectively.

To obtain the LCOE, the energy cost obtained by the wind turbines in each location by different alternatives has been calculated. The energy produced has been estimated as a function of the wind distribution and the characteristic power curve of the selected 10 MW turbine.

**Figure 10:**
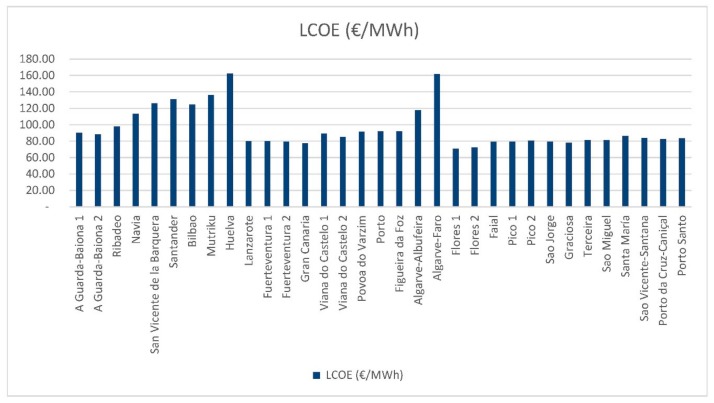
LCOE results.

The LCOE comparison offers information on what might be the best locations for an offshore wind farm without taking into account the parameter of the electricity tariff, only the annual electricity production. As Figure 10 shows, Huelva (Spain) and Algarve-Faro (Portugal), have been the locations with the highest LCOE, exceeding 160 €/MWh.

The locations that have shown the best results have been the Canary Islands, Azores and the Madeira Islands. The lowest LCOE of all is Flores 1 (Azores) with 71.04 €/MWh. Given that the LCOE does not depend on the tariff, these results are maintained for all alternatives proposed.

**Figure 11:**
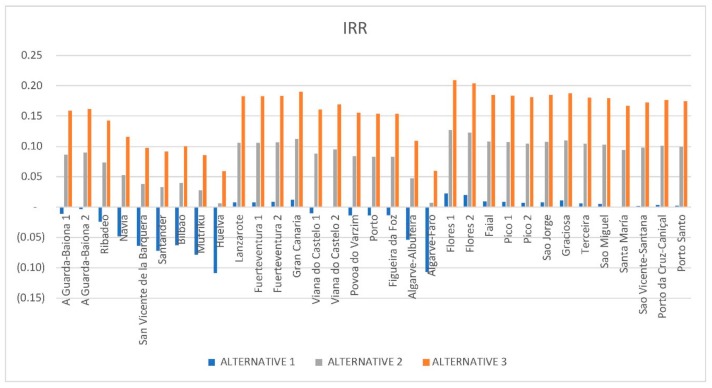
Internal rate of return (IRR) results.

Figure 11 shows how the IRR increases when using a higher electricity tariff. Alternative 2 (e_rate = 100 €/MWh) implies an increase in the IRR by approximately 0.1 points with respect to the values obtained in Alternative 1 (e_rate = 50 €/MWh). Alternative 3 (e_rate = 150 €/MWh) implies an increase in the IRR by approximately 0.07 points with respect to the values obtained in Alternative 2 (e_rate = 100 €/MWh). Comparing these results with the opportunity cost considered in this study (4.92%), Alternative 1 does not offer viability to any location because the results of IRR are lower than 4.92% of the capital cost considered. Alternative 2 would be accepted in terms of IRR for almost all locations, except some in Spain: Santander, Bilbao, Huelva, Mutriku. Projects that consider Alternative 3 give a higher return than the minimum required for all locations.

For the pessimistic scenario, the IRR only reaches positive values in the locations mentioned above that obtained lower LCOE values.

**Figure 12:**
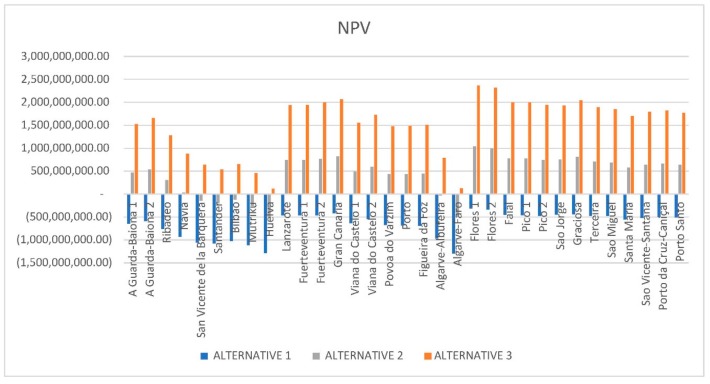
Net present value (NPV) results.

The influence of the variation of the electricity rate on the NPV has also been taken into account in this study and for the locations analyzed. The farm will be economically feasible if the NPV is higher than zero. All the results obtained from NPV have been negative for the pessimistic scenario (see Figure 12). However, the increase in the rate considered for alternative 2 with respect to alternative 1 allows many locations to reach positive values: A Guarda-Baiona 1 and 2, Ribadeo, Canary Islands, Azores and Madeira Islands, etc.

When a higher rate is considered for alternative 3 (e_rate = 150 €/MWh), NPV values increase significantly. All locations are positives now, and some of them exceed €2 billion, such as Gran Canaria (Canary Islands), Graciosa, Flores 1 and 2 (Azores).

## 6. Conclusions

This paper has analyzed economically a concrete offshore wind floating platform in the Atlantic Arc of Europe. The life-cycle cost of a concrete floating platform has been considered to calculate the main economic parameters. It is based on the life-cycle phases of the farm: Conception and definition, design and development, manufacturing, installation, exploitation and dismantling. Moreover, the economic parameters calculated are: Levelized cost of energy (LCOE), internal rate of return (IRR) and net present value (NPV). Their analysis determines the feasibility of the offshore farm.

The study considers the concrete floating offshore wind platform Telewind^®^, a spar platform with a 10 MW offshore wind generator.

In addition, the study analyzes thirteen locations in Spain (the Iberian Peninsula and the Canary Islands) and twenty in Portugal (the Iberian Peninsula, the Azores Islands, and the Madeira Islands).

The lowest LCOE of all is Flores 1 (Azores) with 71.04 €/MWh. This location is also the best IRR result for the pessimistic scenario (with an electric tariff of 50 €/MWh), giving 0.02%. In general, island locations have obtained better economic parameters. For alternative 1, NPV in Bilbao is 1,025,288,577 €. Nevertheless, Fuerteventura 2 (Canary Islands) and Porto da Cruz-Caniçal (Madeira) is 464,581,509 € and 497,607,426 € respectively.

Therefore, results show the most suitable economic area to install a floating offshore wind farm composed of concrete platforms in the South Atlantic area of Europe (Spain and Portugal) that are the Canary Islands for Spain and Flores for Portugal. These places can represent the future developments in offshore wind industry.

## Figures and Tables

**Table 1:** Decommissioning costs (% of C_4_). Source: [15].

Description	Percentage of C_4_
Complete wind turbine—floating	70%
Electric cables	10%
Substation	90%
Mooring	90%

**Table 2:** Characteristics of the Telewind^®^ platform. Source: Data courtesy of Esteyco.

Characteristics	Value	Units
Water depth	110	m
Hub height above MSL	119	m
Wind turbine power	10	MW
Metacentric height in place (GM)	>3	m
Metacentric height transport (GM)	>2	m
Tilt static angle	<10°	°
Overall heave period (T3)	>30	s
Overall pitch period (T3)	>30	s

**Table 3:** Coordinates of the locations.

Location		Coordinates	
SPAIN	A Guarda-Baiona 1	41.86 N	9.18 W
	A Guarda-Baiona 2	41.86 N	9.32 W
	Ribadeo	43.83 N	7.33 W
	Navia	43.63 N	6.53 W
	San Vicente de la Barquera	43.56 N	4.22 W
	Santander	43.57 N	3.66 W
	Bilbao	43.67 N	3.00 W
	Mutriku	43.39 N	2.33 W
	Huelva	36.76 N	7.30 W
CANARY ISLANDS	Lanzarote	29.22 N	13.74 W
	Fuerteventura 1	28.54 N	14.19 W
	Fuerteventura 2	28.40 N	14.29 W
	Gran Canaria	27.77 N	15.31 W
PORTUGAL	Viana do Castelo 1	41.86 N	9.00 W
	Viana do Castelo 2	41.82 N	9.31 W
	Póvoa do Varzim	41.52 N	9.20 W
	Porto	41.05 N	9.27 W
	Figueira da Foz	40.21 N	9.56 W
	Algarve-Albufeira	36.86 N	8.32 W
	Algarve-Faro	36.79 N	7.68 W
AZORES	Flores 1	39.48 N	31.35 W
	Flores 2	39.31 N	31.25 W
	Faial	38.62 N	28.92 W
	Pico 1	38.27 N	28.49 W
	Pico 2	38.310 N	27.89 W
	Sao Jorge	38.67 N	27.85 W
	Graciosa	39.01 N	28.14 W
	Terceira	38.51 N	26.66 W
	Sao Miguel	37.90 N	25.63 W
	Santa María	37.04 N	25.00 W
MADEIRA ISLANDS	Sao Vicente-Santana	32.89 N	16.99 W
	Porto da Cruz-Caniçal	32.82 N	16.68 W
	Porto Santo	33.11 N	16.44 W

**Table 4:** Main characteristics of the locations selected.

Location		Distance Farm-Shore (m)	Distance Farm-Onshore Facilities (m)	Depth (m)
SPAIN	A Guarda-Baiona 1	8	35.2	150
	A Guarda-Baiona 2	35.6	55.7	500
	Ribadeo	17.6	89.5	150
	Navia	10.3	48.7	150
	San Vicente de la Barquera	14.1	40.7	500
	Santander	8	15.8	400
	Bilbao	18.5	25.6	700
	Mutriku	9.7	60.7	400
	Huelva	29.5	93	500
CANARY ISLANDS	Lanzarote	9.6	76	800
	Fuerteventura 1	12.8	73.2	800
	Fuerteventura 2	13.6	87	500
	Gran Canaria	8.2	9.7	400
PORTUGAL	Viana do Castelo 1	12	12.7	100
	Viana do Castelo 2	27.8	28.5	150
	Póvoa do Varzim	20	30.4	100
	Porto	33.1	33.1	150
	Figueira da Foz	51	62.1	150
	Algarve-Albufeira	20.3	36.3	100
	Algarve-Faro	20.3	26.1	600
AZORES	Flores 1	2	17	250
	Flores 2	2.3	5	300
	Faial	1.8	28	1000
	Pico 1	12.2	19	1000
	Pico 2	7	42	500
	Sao Jorge	1	38	700
	Graciosa	2.3	11	500
	Terceira	16	15	700
	Sao Miguel	1.7	47	700
	Santa María	3	19	500
MADEIRA ISLANDS	Sao Vicente-Santana	1.8	38	500
	Porto da Cruz-Caniçal	2.8	17.4	200
	Porto Santo	1	13	80

**Table 5:** Alternatives based on the electric tariff considered (€/MWh).

ALTERNATIVE	Electric tariff (€/MWh)
ALTERNATIVE 1	50
ALTERNATIVE 2	100
ALTERNATIVE 3	150
